# It takes a village: Fixed-effects analysis of neighborhood collective efficacy and children's development

**DOI:** 10.1016/j.je.2016.08.018

**Published:** 2017-07-05

**Authors:** Kayoko Ichikawa, Takeo Fujiwara, Ichiro Kawachi

**Affiliations:** a Department of Health Informatics, Kyoto University School of Public Health, Kyoto, Japan; b Department of Social Medicine, National Research Institute for Child Health, Tokyo, Japan; c Department of Social and Behavioral Sciences, Harvard T.H. Chan School of Public Health, Boston, MA, USA

**Keywords:** Collective efficacy, Social capital, Child development, Fixed-effects model

## Abstract

**Background:**

Previous studies suggest that neighborhood social capital is associated with children's mental health. The purpose of this study was to examine the association between neighborhood collective efficacy and children's psychosocial development.

**Methods:**

We used data on children and their parents (*n* = 918) who were part of the Japanese study of Stratification, Health, Income, and Neighborhood (JSHINE) from 2010 to 2013 (wave 1 and wave 2). Households were recruited from the Tokyo metropolitan area through clustered random sampling. Changes in children's psychosocial development (assessed using a child behavioral checklist) between waves 1 and 2 were regressed on parents' perceptions of changes in neighborhood collective efficacy (social cohesion and informal social control).

**Results:**

Change in perception of neighborhood social cohesion was inversely associated with change in child total problems (*β* = −0.22; 95% confidence interval [CI]: −0.37 to −0.001; effect size *d* = −0.03). Change in perceptions of neighborhood informal social control was inversely associated with change in children's externalizing problems (*β* = −0.16; 95% CI: −0.30 to −0.03; *d* = −0.02).

**Conclusions:**

The results of these fixed-effects models suggest that strengthening neighborhood collective efficacy is related to improvements in child psychosocial development.

## Highlights

• The fixed-effects model can control for time-invariant confounding factors.
• Neighborhood social cohesion was inversely associated with child total problems.
• Neighborhood informal social control was inversely associated with child problems.
• Neighborhood collective efficacy prevents child psychosocial problems.

## Introduction

Previous studies indicate that neighborhood social capital influences child development and health.^1^, ^2^, ^3^, ^4^, ^5^, ^6^, ^7^ Three different mechanisms have been postulated: i) the institutional resources model, which posits that neighborhoods with higher stocks of social capital are endowed with higher functioning institutions (e.g., because of more intense parental involvement in local schools); ii) the relationship model, which posits that high social capital neighborhoods have more supportive relationships between residents, which support the nurturing of children; and iii) the norms and collective efficacy model, which posits that neighborhoods with high social capital are better able to enforce pro-social norms and are more willing to intervene for the common good.^8^ The concept of collective efficacy — proposed by Sampson, Earls, and Raudenbush — is operationalized as the combination of two neighborhood characteristics: social cohesion (i.e., levels of trust between residents) and informal social control (i.e., the ability of adults in the neighborhood to supervise the development of children).

Following Sampson's seminal study in 1997 concerning the relation between neighborhood collective efficacy and crime victimization,^9^, ^10^ subsequent studies have linked the concept to children's mental health.^4^, ^6^, ^8^, ^11^, ^12^ However, empirical studies to date have been primarily cross-sectional in design and unable to establish the causal nature of the relation between neighborhood collective efficacy and child health outcomes.^13^ Experimental and quasi-experimental methods are needed to identify the causal relations between collective efficacy and child psychosocial development. Accordingly, we sought to test the association between neighborhood collective efficacy and children's psychosocial development, taking advantage of a fixed-effects model, which can control for time invariant unobserved and observed confounding characteristics.

## Methods

### Participants

We used the baseline and second survey waves of the ongoing Japanese study of Stratification, Health, Income, and Neighborhood (J-SHINE) cohort study established in 2010. Details of the study have been previously described.^14^ Briefly, the baseline survey (wave 1) was carried out in 2010–2011 (adults participants in 2010 and their children in 2011), when a clustered random sample of individuals aged 25–50 years residing in four municipalities in urban or suburban settings of the Tokyo metropolitan area were invited to participate. The household survey inquired about the health of all children under the age of 18 years co-residing with the subjects. A follow-up survey (wave 2) was conducted in 2012–2013 (adults in 2012 and their children in 2013). In wave 1, 13,920 individuals were randomly selected from the *koseki* registration system, a compulsory domiciliary registration system throughout Japan. Of the individuals invited to participate, 4385 men and women responded (31.6% response rate, which is typical for surveys of community-dwelling adults). The number of households with children was 2244, and 1520 (67.7%) of these households, including 2710 children under 18 years and over 4 years old, agreed to participate in the baseline children's survey. The follow-up survey was administered to the same individuals. Of the 1520 wave 1 households, 1121 households, including 1887 children, responded to the wave 2 survey (follow up rate = 73.8%).

**Fig. 1:**
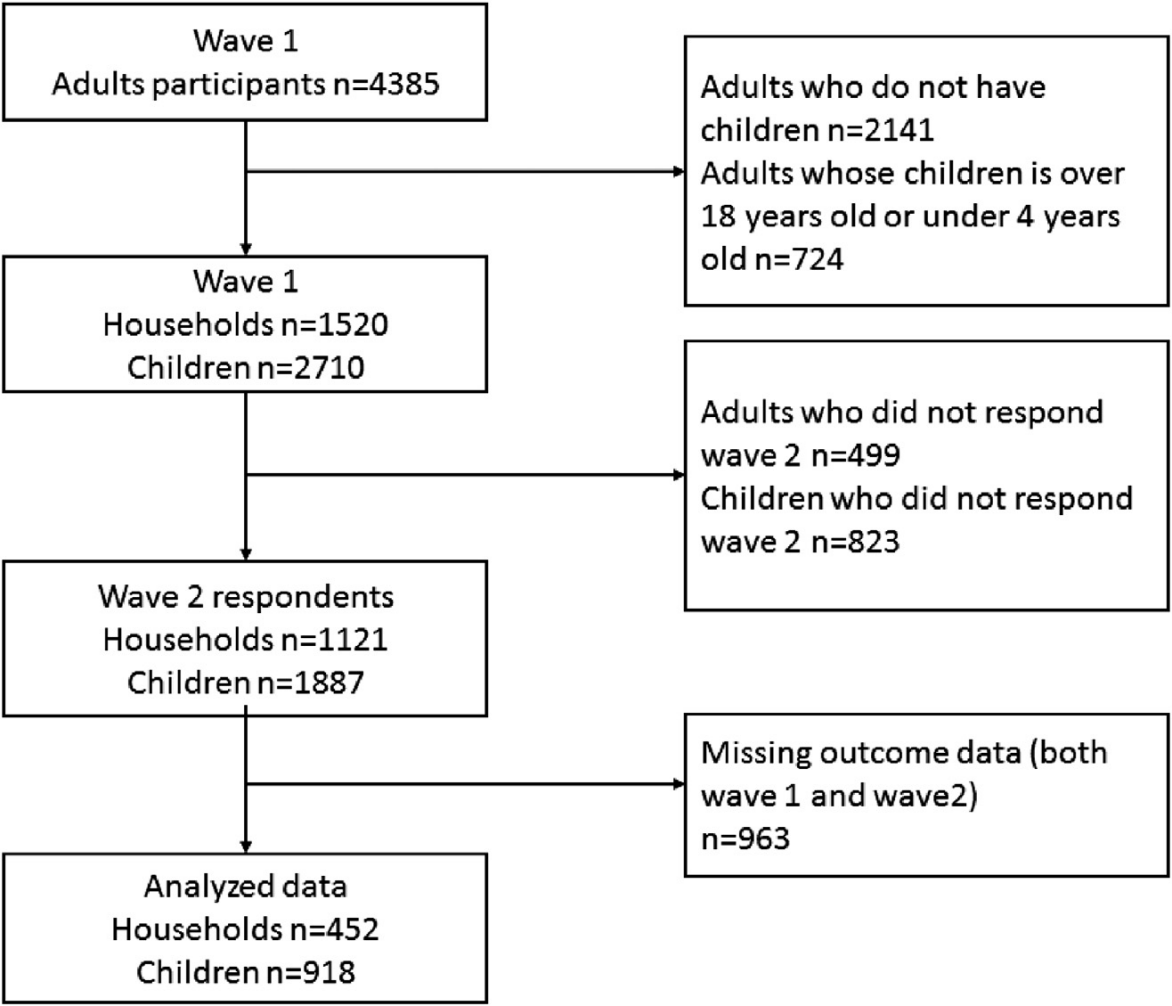
Participants' flow chart.

After excluding children for whom we did not have complete outcome information at both waves, we were left with a final analytic sample of 918 children (452 households) (Fig. 1).

The J-SHINE was conducted using computer-assisted personal interviewing, unless the participants requested a face-to-face interview. The study protocol and informed consent were approved by the ethics committee of the Graduate School of Medicine of the University of Tokyo.

### Measurements

#### Exposure: change in collective efficacy between wave 1 and wave 2

Social capital was assessed using questions asked to parents relating to perceptions about their neighborhood. Based on principal component analysis, we categorized 10 items on the survey into two sub-scales: social cohesion and informal social control.

The social cohesion subscale was made up of five items asking respondents how strongly they agreed that “people around here are willing to help neighbors,” “this is a close-knit neighborhood,” “people in this neighborhood can be trusted,” “people in this neighborhood generally don't get along with each other,” and “people in this neighborhood do not share the same values” (the last two statements were reverse coded) (Cronbach alpha = 0.79). Informal social control was made up of five items asking respondents about how confident they were that adults in the neighborhood could be counted on intervene if: (1) children were skipping school and hanging out on a street corner, (2) children were spray-painting graffiti on a local building, (3) children were showing disrespect to an adult, (4) a fight broke out in front of their house, or (5) a community hall close to their home was threatened with budget cuts (Cronbach alpha = 0.87). All responses were coded on a five point Likert-type scale (“Would you say it is very likely, likely, neither likely nor unlikely, unlikely, or very unlikely?”) and summed. Higher scores indicate higher collective efficacy.

#### Outcome: change in the child behavior check list 4–18 between wave 1 and wave 2

Our outcome variable, children's psychosocial developmental problems, was assessed with the Child Behavior Check List 4–18 (CBCL4–18), which targets children aged 4–18 years.^15^, ^16^ This scale provides a score for total behavior problems, as well as for the composite internalizing and externalizing behavior problems. The internalizing problem sub-scale includes physical problems, withdrawal, and anxiety problems, while the externalizing problem sub-scale is made up of aggression and delinquency. The remaining subscales assess social problems, cognitive problems, and attention problems. Ratings were completed by the caregivers (the mother, in 60.5% of cases [555/917]). The *T* score of each CBCL scores was calculated using the standardized distribution among Japanese children, where the mean score represents the 50th percentile. The checklist has been shown to have good reliability and validity.^15^, ^16^, ^17^ Change in CBCL4–18 scores of children from wave 1 to wave 2 was used as the outcome of this study.

### Statistical analysis and covariates

We conducted fixed-effects regression to control for time-invariant observed and unobserved confounding variables. We measured parents' age and educational attainment, children's sex and age, duration of residence, household income, and number of family members in wave 1 as observed time-invariant variables. We controlled for time-varying covariates between waves, including change in family income, change of job, residential moves, change in number of family members, as well as change in the health status of family members. The fixed-effects models were conducted with generalized liner regressions for estimating the association between changes in collective efficacy and changes in child development. We further addressed clustering at the area level using multilevel analysis. We conducted our analyses sequentially, first constructing unadjusted models and then adjusting for time-varying variables.

All analyses controlled for clustering by family and city. Missing variables about age of parents and children were imputed by the mean of each age. Missing data about categorical variables of parent's education, job, income, number of family members, children's sex, and living year were treated as dummy variables. All analyses were performed with STATA 13.0 (Stata Corp, College Station, TX, USA).

## Results

Table 1 shows the demographic characteristics of the children and family in this study. The average age was 41 years among responding mothers, 42 years among responding fathers, and 9.5 years among the children. Approximately half of the participating children were boys; 73% of the parents had more than high school education; and 62% of the families had lived in their neighborhoods for more than 5 years, while 4% of families moved residences between 2010 (wave 1) and 2012 (wave 2).

**Table 1:** Individual-level demographic characteristics of children and families in wave 1 and wave 2 (*n* = 918 children).

Variables	Wave 1	Wave 2
*Area characteristics*		
Tokyo urban area, Adachi	173 (18.9)	
Tokyo urban area, Mitaka	179 (19.5)	
Tokyo suburban area, Kashiwa	326 (35.5)	
Tokyo suburban area, Tokorozawa	240 (26.1)	

*Family characteristics*		
Mother's age, years, mean (SD)	40.8 (9.7)	
Father's age, years, mean (SD)	41.5 (7.0)	
Mother's education		
>12 years	666 (72.6)	
Unknown	19 (2.1)	
Father's education		
>12 years	668 (72.3)	
Unknown	37 (4.0)	
Working mother	493 (53.7)	
Working father	812 (88.5)	
Numbers of family		
≤4	612 (66.7)	
Unknown	2 (0.2)	
Family income		
<JPY 5 million^a^	187 (20.4)	
JPY 5–7.5 million	279 (30.4)	
JPY 7.5–10 million	185 (20.2)	
>JPY 10 million	186 (20.3)	
Unknown	81 (8.8)	
Domestic violence	290 (31.6)	
Duration of residence		
≤5 years	338 (36.8)	
6–10 years	284 (30.9)	
>10 years	291 (31.7)	
Unknown	5 (0.5)	
No social network with neighborhood	464 (50.5)	

*Children's characteristics*		
Sex, male	457 (49.8)	
Age, years		
4–7	345 (37.6)	
8–11	321 (35.0)	
12–17	252 (27.5)	

*Change from wave 1 to wave 2*		
Moving		36 (3.9)
Job		281 (30.6)
Family income		
Down		84 (9.1)
Same		793 (86.3)
Up		42 (4.6)
Family expenditure		
Down		6 (0.7)
Same		830 (90.3)
Up		83 (9.0)
Health status of responders		133 (14.5)
Health status of family members		90 (9.8)
Family structure		60 (6.5)
Newborn		43 (4.7)

*Collective efficacy*		
Responder, mother	555 (60.5)	555 (60.5)
Social trust, mean (SD)	16.3 (2.9)	16.8 (2.9)
Informal social control, mean (SD)	16.1 (3.6)	16.3 (3.5)

*CBCL4–16 T score, mean (SD)*		
Physical problem	52.6 (5.2)	52.4 (4.9)
Social problem	53.5 (5.3)	53.2 (5.0)
Thought problem	51.2 (4.1)	52.8 (4.9)
Delinquency	53.4 (5.3)	52.5 (4.8)
Withdrawal	53.6 (5.3)	53.3 (5.0)
Anxiety problem	52.9 (4.8)	52.7 (4.7)
Attention problem	53.2 (5.3)	52.8 (4.9)
Aggression	53.6 (5.5)	53.2 (5.2)
Internalizing problem	50.6 (7.0)	50.2 (6.9)
Externalizing problem	50.4 (7.9)	49.6 (7.4)
Total problem	49.7 (9.1)	48.3 (9.2)

CBCL, child behavior checklist; SD, standard deviation.

Values reported as *n* (%), unless otherwise noted.

a JPY = Japanese Yen, JPY 120 is approximately equal to US $1.

Table 2 describes the association between change in parental perceptions of neighborhood social cohesion and children's development between wave 1 and wave 2. In the unadjusted model, each standard deviation (SD) increment in social cohesion was inversely associated with the child total problem score (standard coefficient [*β*] = −0.19; 95% CI: −0.37 to −0.002; effect size of Cohen's *d* [*d*] = −0.03). Adjusted for time-varying confounding variables, each SD increment in social cohesion remained significantly associated with a decrease in child total problem score (*β* = −0.22; 95% CI: −0.37 to −0.001; *d* = −0.03).

**Table 2:** Fixed-effects models of the association between parent's social cohesion and children's psychosocial development (*n* = 907).

CBCL *T* score	Fixed-effects model, unadjusted		Fixed-effects model, adjusted^a^	
	*β*	95% CI	*β*	95% CI
Physical problem	−0.03	−0.17, 0.12	−0.01	−0.15, 0.13
Social problem	−0.04	−0.15, 0.07	−0.05	−0.16, 0.06
Thought problem	0.01	−0.09, 0.12	0.02	−0.08, 0.13
Delinquency	−0.05	−0.18, 0.08	−0.05	−0.18, 0.08
Withdrawal	−0.07	−0.19, 0.06	−0.07	−0.20, 0.05
Anxiety problem	−0.04	−0.15, 0.08	−0.03	−0.14, 0.08
Attention problem	−0.09	−0.20, 0.02	−0.09	−0.20, 0.02
Aggression	−0.07	−0.19, 0.06	−0.07	−0.20, 0.05
Internalizing problem	−0.11	−0.27, 0.05	−0.12	−0.28, 0.04
Externalizing problem	−0.07	−0.23, 0.09	−0.06	−0.23, 0.10
Total problem	**−0.19**	**−0.37, −0.002**	**−0.19**	**−0.37, −0.001**

CBCL, Child Behavior Checklist; CI, confidence interval.

The bold values means statistically significant.

a Adjusted by time-variant variables between wave 1 and wave 2 (family income and outcome, job, moving, family member's change, health status of family), clustered by family ID.

Table 3 describes the association between change in parental perceptions of neighborhood informal social control and their children's development between waves 1 and 2. In the unadjusted model, each SD difference in informal social control was associated with lower children's aggression scores (*β* = −0.11; 95% CI: −0.21 to −0.01; *d* = −0.02), as well as externalizing problems (*β* = −0.17; 95% CI: −0.30 to −0.03; *d* = −0.03). Adjusted for time-variant variables, informal social control remained significantly associated with children's externalizing problems (*β* = −0.16; 95% CI: −0.30 to −0.03; *d* = −0.02).

**Table 3:** Fixed-effects models of the association between parent's informal social control and children's psychosocial development (*n* = 903).

CBCL *T* score	Fixed-effects model, unadjusted		Fixed-effects model, adjusted^a^	
	*β*	95% CI	*β*	95% CI
Physical problem	0.00	−0.12, 0.11	0.01	−0.11, 0.13
Social problem	−0.02	−0.12, 0.07	−0.02	−0.11, 0.07
Thought problem	−0.03	−0.12, 0.06	−0.03	−0.12, 0.06
Delinquency	−0.06	−0.16, 0.05	−0.06	−0.16, 0.05
Withdrawal	−0.06	−0.16, 0.04	−0.05	−0.15, 0.05
Anxiety problem	−0.02	−0.12, 0.07	−0.01	−0.10, 0.09
Attention problem	−0.02	−0.11, 0.08	−0.01	−0.10, 0.09
Aggression	**−0.11**	**−0.21, −0.01**	−0.10	−0.20, 0.01
Internalizing problem	−0.06	−0.20, 0.07	−0.05	−0.18, 0.08
Externalizing problem	**−0.17**	**−0.30, −0.03**	**−0.16**	**−0.30, −0.03**
Total problem	−0.09	−0.25, 0.06	−0.09	−0.24, 0.07

CBCL, child behavior checklist; CI, confidence interval.

The bold values means statistically significant.

a Adjusted by change between wave 1 and wave 2 about family income and outcome, job, moving, family member's change, health status of family, clustered by family ID.

eTable 1–4 show how social cohesion was associated with a decrease in child total problem score, while informal social control was associated with lower externalizing problems, especially for boys.

## Discussion

This study examined the longitudinal association between change in neighborhood collective efficacy (as perceived by parents) and change in children's psychosocial development. By accounting for time-invariant observed and unobserved confounding factors, our results lend credence to the notion that neighborhood social capital promotes child developmental outcomes. In detail, social cohesion was associated with a decrease in child total problem scores, while informal social control was associated with lower externalizing problems, especially for boys. For children's healthy development, people living in urban cities that do not have strong social ties should promote more neighborhood social cohesion and informal social control. The characteristics of the areas sampled in our study (Tokyo urban and suburban areas) are typical of Japanese urban cities. Many young people come from other prefectures to marry in Tokyo and raise children in an urban or suburban area. Therefore, social ties are typically weaker than in rural areas.

Our findings also corroborate the findings of previous observational studies, which found that neighborhood collective efficacy was inversely associated with levels of antisocial behavior at school entry,^18^ as well as less violent attitudes among adolescents.^19^ As expressed by the frequently repeated African proverb, “it takes a village to raise a child”, there is plausibility in the notion that the socialization of children depends not just on parental involvement within the family, but also on the collective efforts of adults within the community.

Although many previous cross-sectional studies suggested an association between community collective efficacy and child mental health, to the best to our knowledge, this is the first study to examine child developmental outcomes using a fixed-effects approach. Although one similar previous study^20^ assessed the stability of collective efficacy over time (4 years) using a fixed-effect model and revealed the association between collective efficacy and mental health using multilevel analysis in cross-sectional data, it did not directly examine the association between collective efficacy and health. Although the effect sizes of the change in child total problems (Cohen's *d* = −0.03) and the change in children's externalizing problems (*d* = −0.02) by collective efficacy were small in this study, this research revealed the causality of collective efficacy and children's psychosocial development.

Some limitations should be noted. First, fixed-effects models can only account for time-invariant variables. Residual confounding is still possible from the influence of unobserved time-varying variables, such as domestic violence (DV) status. Although we did not update DV status at wave 2 and therefore we could track changes in DV status between wave 1 and wave 2, DV status may not be easy to change, thus the impact of DV and child behavior problem is limited. Second, both the exposure and outcomes in our analysis depended on parental responses to the survey, thereby raising the possibility of common source bias. On the other hand, if the parental assessments were confounded by time-invariant characteristics (such as parental personality), they ought to have been accounted for in the fixed-effects models. Third, the first-differencing approach (regressing the change in outcome on change in exposure) cannot rule out reverse causation (i.e., the possibility that improvements in child behavior over time resulted in improvements in parental assessments of neighborhood social capital). Fourth, the follow-up period between waves was relatively short (2 years), which may under-estimate the effect size of social capital on developmental outcomes. Further follow-up is desirable to confirm out findings. Last, we restricted our analyses to the participants who responded to both waves of the survey, so roughly half of the original sample (*n* = 954) were dropped. However, our comparison of the analytic sample with the dropped cases revealed minor differences in their baseline characteristics.

The implications of our findings — if they are borne out — suggest that strengthening community collective efficacy may promote healthy child development, especially in urban areas with weaker social ties. Although we could not measure school-based effects, examples of potential interventions include strengthening neighborhood associations, such as “omatsuri (traditional neighborhood-based festivals)” and “toukou mimamori (community volunteers who supervise children walking to school)”. Future experimental studies are needed in this area.

## Conflicts of interest

None declared.
