# Supplementary material for: It takes a village: Fixed-effects analysis of neighborhood collective efficacy and children's development
Source: J Epidemiol. 2017 Jul 5;27(8):368–72. doi: 10.1016/j.je.2016.08.018 (PMC5549262; doi:10.1016/j.je.2016.08.018)
Supplement: Supplementary file 1 [file mmc1.pdf]

eTable 1. Fixed-effects models of the association between parent's social cohesion and their boy's psychosocial development (n=453)

	Fixed-effects model, unadjusted		Fixed-effects model, adjusted[a]	
CBCL T score	β	95% CI	β	95% CI
Physical problem	−0.02	−0.22, 0.19	−0.03	−0.23, 0.18
Social problem	−0.07	−0.22, 0.08	−0.08	−0.23, 0.07
Thought problem	0.03	−0.12, 0.18	0.01	−0.14, 0.16
Delinquency	−0.17	−0.35, 0.01	−0.16	−0.34, 0.01
Withdrawal	−0.10	−0.29, 0.08	−0.12	−0.30, 0.07
Anxiety problem	−0.07	−0.24, 0.10	−0.08	−0.24, 0.09
Attention problem	−0.12	−0.27, 0.04	−0.12	−0.27, 0.03
Aggression	−0.08	−0.27, 0.10	−0.09	−0.28, 0.10
Internalizing problem	−0.09	−0.32, 0.14	−0.11	−0.34, 0.12
Externalizing problem	0.03	−0.20, 0.26	0.03	−0.20, 0.25
Total problem	−0.11	−0.37, 0.15	−0.13	−0.38, 0.13

CBCL, Child Behavior Checklist; CI, confidence interval.

[a] adjusted by time-variant variables between wave 1 and wave 2 (family income and outcome, job, moving, family member's change, health status of family), clustered by family ID

eTable 2. Fixed-effects models of the association between parent's social cohesion and their girl's psychosocial development (n=450)

	Fixed-effects model, unadjusted		Fixed-effects model, adjusted[a]	
CBCL T score	β	95% CI	β	95% CI
Physical problem	−0.04	−0.22, 0.14	−0.01	−0.19, 0.17
Social problem	0.00	−0.16, 0.17	−0.01	−0.17, 0.16
Thought problem	−0.01	−0.15, 0.14	0.03	−0.11, 0.18
Delinquency	0.06	−0.11, 0.23	0.05	−0.13, 0.22
Withdrawal	−0.01	−0.17, 0.15	−0.01	−0.17, 0.14
Anxiety problem	0.01	−0.15, 0.16	0.03	−0.12, 0.19
Attention problem	−0.07	−0.22, 0.09	−0.07	−0.22, 0.09
Aggression	−0.05	−0.21, 0.11	−0.07	−0.23, 0.09
Internalizing problem	−0.13	−0.34, 0.09	-	-
Externalizing problem	−0.15	−0.37, 0.07	−0.15	−0.37, 0.08
Total problem	−0.24	−0.50, 0.01	−0.24	−0.49, 0.02

CBCL, Child Behavior Checklist; CI, confidence interval.

[a] adjusted by time-variant variables between wave 1 and wave 2 (family income and outcome, job, moving, family member's change, health status of family), clustered by family ID

eTable 3. Fixed-effects models of the association between parent's informal social control and their boy's psychosocial development (n=453)

	Fixed-effects model, unadjusted		Fixed-effects model, adjusted[a]	
CBCL T score	β	95% CI	β	95% CI
Physical problem	0.08	−0.08, 0.25	0.09	−0.07, 0.26
Social problem	0.01	−0.11, 0.13	0.00	−0.12, 0.12
Thought problem	0.02	−0.10, 0.14	0.02	−0.11, 0.14
Delinquency	−0.12	−0.27, 0.02	−0.13	−0.27, 0.01
Withdrawal	−0.10	−0.25, 0.05	−0.11	−0.26, 0.04
Anxiety problem	0.01	−0.13, 0.14	0.01	−0.13, 0.14
Attention problem	−0.03	−0.16, 0.10	−0.02	−0.15, 0.10
Aggression	−0.09	−0.25, 0.07	−0.09	−0.25, 0.07
Internalizing problem	−0.01	−0.20, 0.18	−0.02	−0.21, 0.16
Externalizing problem	**−0.19**	**−0.37, 0.00**	**−0.21**	**−0.39, −0.03**
Total problem	−0.09	−0.30, 0.12	−0.12	−0.33, 0.09

CBCL, Child Behavior Checklist; CI, confidence interval.

[a] adjusted by change between wave 1 and wave 2 about family income and outcome, job, moving, family member's change, health status of family, clustered by family ID

eTable 4. Fixed-effects models of the association between parent’s informal social control and their girl's psychosocial development (n=447)

	Fixed-effects model, unadjusted		Fixed-effects model, adjusted[a]	
CBCL T score	β	95% CI	β	95% CI
Physical problem	−0.10	−0.26, 0.05	−0.08	−0.23, 0.07
Social problem	−0.07	−0.21, 0.07	−0.06	−0.20, 0.07
Thought problem	−0.09	−0.22, 0.04	−0.09	−0.21, 0.04
Delinquency	0.02	−0.13, 0.16	0.02	−0.13, 0.17
Withdrawal	−0.02	−0.16, 0.11	0.01	−0.13, 0.14
Anxiety problem	−0.05	−0.18, 0.08	−0.03	−0.16, 0.10
Attention problem	−0.01	−0.14, 0.12	0.00	−0.14, 0.13
Aggression	−0.12	−0.26, 0.01	−0.10	−0.23, 0.03
Internalizing problem	−0.12	−0.30, 0.06	−0.09	−0.27, 0.09
Externalizing problem	−0.14	−0.33, 0.04	**−0.12**	**−0.31, 0.07**
Total problem	−0.09	−0.31, 0.12	−0.07	−0.28, 0.15

CBCL, Child Behavior Checklist; CI, confidence interval.

[a] adjusted by change between wave 1 and wave 2 about family income and outcome, job, moving, family member's change, health status of family, clustered by family ID
